# Cold Atmospheric Plasma Conveys Selectivity Against Hepatocellular Carcinoma Cells *via* Triggering EGFR(Tyr1068)-Mediated Autophagy

**DOI:** 10.3389/fonc.2022.895106

**Published:** 2022-07-04

**Authors:** Danjun Wang, Jianying Zhang, Linhan Cai, Xiaofeng Dai

**Affiliations:** ^1^ Beijing Genomics Institution (BGI) College & Henan Institute of Medical and Pharmaceutical Sciences in Academy of Medical Science, Zhengzhou University; ^2^Wuxi School of Medicine, Jiangnan University, Wuxi, China; ^3^CAPsoul Medical Biotechnology Company, Ltd., Beijing, China

**Keywords:** cold atmospheric plasma, autophagy, post-translational modification, EGFR, hepatocellular carcinoma

## Abstract

Hepatocellular carcinomas remain as a global health threat given its high mortality rate. We have previously identified the selectivity of cold atmospheric plasma (CAP) against multiple types of malignant tumors and proposed it as a promising onco-therapeutic strategy. Here, we investigated its roles in controlling hepatocellular carcinoma malignancy and one possible driving molecular mechanism. By focusing on post-translational modifications including acetylation, phosphorylation, and ubiquitination, we identified the crosstalk between EGFR acetylation and EGFR(Tyr1068) phosphorylation and their collective roles in determining LC3B ubiquitination and proposed the EGFR/p-JNK/BIRC6/LC3B axis in CAP-triggered autophagy. Our study not only demonstrated the selectivity of CAP against hepatocellular carcinoma malignancy and confirmed its roles as an onco-therapeutic tool but also opened the horizon of translating CAP into clinics toward a broader scope that included human longevity and anti-aging.

## Introduction

According to the latest Global Cancer Statistics, liver cancer contributes to the 3rd leading cause of cancer death and is ranked as the second mortality among men in 2020 (1). Primary liver cancers are largely comprised of hepatocellular carcinomas (HCCs) and intrahepatic cholangiocarcinomas (ICCs), each accounting for approximately 80% and 15% of the incidence (2, 3). A small percentage of patients [ranging from 0.4% and 14.2% (4, 5)] harbor the hybrid of both subtypes, namely, cHCC-ICC (6). Being the predominant subtype of primary liver cancers, HCC remains as a major global health threat and thus become the focus of this study.

The prognosis of liver cancers is, in general, poor, with the median survival time of HCC being approximately 3 months without treatment (7, 8). Surgery is the first-line therapy for HCC treatment (9–11). However, due to a hidden onset, only approximately 20%–30% of patients have the opportunity to receive surgery as most patients have already reached the middle and late stage on diagnosis (10), leaving chemotherapy and immunotherapy still being the mainstream therapeutic choices for HCC (12). In addition to the negative impacts of these therapeutic interventions on the human immune system, HCC relapse after receiving these treatments is almost inevitable due to the high intrinsic heterogeneity of such cancers that can easily lead to the development of differential therapeutic responses (13).

Sorafenib (a kinase inhibitor) was the first drug approved by the Food and Drug Administration (FDA) for HCC treatment that can extend patient survival to no more than 3 months (14, 15). Nivolumab and pembrolizumab, two PD1 immune checkpoint inhibitors, have achieved an unprecedented favorable therapeutic outcome for a small fraction of HCC patients in clinical trials (16, 17), yet no biomarker is currently available for predicting such a therapeutic response. Therefore, establishing novel techniques for effective and safe HCC treatment toward its ultimate eradication represents an urgent global challenge.

Cold atmospheric plasma (CAP), formed by the ionization of gas molecules, belongs to the fourth state of matter following solid, liquid, and gas and is composed of various electronics, ions, atoms, and free radicals (18). We have previously proven that CAP can selectively kill triple-negative breast cancer cells (19–21), bladder cancer cells (22), and prostate cancer cells (23) without observable side effects and now endeavor to investigate its efficacy in resolving HCC and the underlying molecular mechanism.

## Materials and Methods

### Cell Culture

Human hepatocellular carcinoma cell lines Huh7 (Catalog No. CL-0120) and HepG2 (Catalog No. SCSP-510) were purchased from the National Collection of Authenticated Cell Cultures (Shanghai, China). The normal liver cell line LO2 (Catalog No. CL-0111) was purchased from Procell Life Science & Technology Co., Ltd (Wuhan, China).

Huh7 and HepG2 were cultivated in dulbecco's modified eagle medium (DMEM) supplemented with 10% fetal bovine serum (Catalog No. SH30406.05, Cytiva) and 1% penicillin/streptomycin solution (Catalog No. BL505A, Biosharp), in a 37°C incubator supplemented with 5% CO_2_. LO2 was cultivated in RPMI1640 supplemented with 10% fetal bovine serum (Catalog No. SH30406.05, CytivaGibco) and 1% penicillin/streptomycin solution (Catalog No. BL505A, Biosharp).

### siRNA Design

The siRNAs of CBP and BIRC6 (**Supplementary Table 1**) were synthesized by Sai Suofei Biological Technology Co., Ltd (Wuxi, China) and Genwiz Biological Technology Co., Ltd (Suzhou, China), respectively.

### Q-PCR

The TRIzol reagent [Catalog No. DP419, TianGen (Beijing, China)] was used to extract total RNA from a 6-well-plate. 24-48 h after transfection followed by reverse transcription, RNA was converted to cDNA using PrimeScriptRT reverse transcriptase [Catalog No. RR047A, Takara (Japan)]. The qPCR was performed using the Roche LightCycler 480 qPCR system and qPCR kit [Catalog No. CW0957M, Cwbio (Beijing, China)], following the manufacturer’s protocol. The primers are listed in **Supplementary Table 2**. The relative expression level was calculated using the 2^−△△Ct^ method. Student’s t-test was used to access the statistical significance, with p < 0.05 being used as the threshold.

### Western Blot

Total protein was extracted after 24-48 h siRNA transfection or 8 h CAP treatment using an Radio-Immunoprecipitation Assay (RIPA) lysis buffer supplemented with protease and phosphatase inhibitors (Catalog No. P1010, Catalog No. CW2383, CWbio). The BCA Protein Assay Kit [Catalog No. P0010, Beyotime (Shanghai, China)] was used to estimate protein concentration. Protein samples were separated on sodium dodecyl sulfate (SDS) polyacrylamide gel and then transferred to polyvinylidene difluoride (PVDF) membranes that were incubated with primary antibodies overnight at 4°C.

Secondary antibodies were diluted and incubated with the membranes for 1 h at room temperature, followed by washing using Tris-buffered saline with Tween for 3 times with 5 min for each time. The signal was detected by the Tanon-2500B imaging apparatus after the disposal of the WB developer [Catalog No. E412-02-AA/B, Vazyme (Nanjing, China)].

The primary antibodies used in this study include p-EGFR (1068) [(Catalog No. 3777S, Cell Signaling Technology (Boston, America)], , LC3B (Catalog No. 83506S, Cell Signaling Technology), BIRC6 [Catalog No. ab19609, Abcam (Cambridge, UK)], p-JNK (Catalog No. 4668S, Cell Signaling Technology), KI67 (Catalog No. ab16667, Abcam), K48 (Catalog No. 8081S, Cell Signaling Technology), acetyllysine mouse mAb [Catalog No. PTM-102, PTM Biolab(Hangzhou, China)], crotonyllysine mouse monoclonal antibody (mAb) (Catalog No. PTM-502, PTM Biolab), malonyllysine mouse mAb (Catalog No. PTM-902, PTM Biolab), GAPDH [Catalog No. AC001, Proteintech (Chicago, America)]. The secondary antibodies used include Horseradish Peroxidase (HRP)-conjugated anti-rabbit immunoglobin G (IgG) (Catalog No. A0208, Beyotime) and anti-mouse IgG (Catalog No. A0216, Beyotime).

### Immunofluorescence

Cells were slightly washed by Phosphate Buffered Saline (PBS) for 3 times, where they were supplemented with 4% pre-chilled paraformaldehyde [Catalog No. P1110, Solarbio (Beijing, China)] and stewed for 15 min before immobilization. Cells were permeabilized by PBS supplemented with 0.5% Triton X-100 [Catalog No. 9002-93-1, MACKLIN (Shanghai, China)].

Cells were blocked using 5% bovine serum albumin (Catalog No. 0332-100G, Amresco) for 30 min at room temperature followed by the removal of a non-specific combination. Cells were incubated with the primary antibody LC3B (Catalog No. 83506S, Cell Signaling Technology) at 4°C overnight. Cells were washed with PBST (PBS added with 5% Tween) (Catalog No. T8220, Solarbio) and incubated with the secondary antibody goat anti-rabbit IgG Heavy and light chains of immunoglobulins (H&L) (Alexa Fluor^®^ 488) [Catalog No. abs20025, Absin (Shanghai, China)].

After incubating cells with the secondary antibodies, the washing process was repeated for 3 times with PBST. The nuclei were counterstained by 4',6-diamidino-2-phenylindole (DAPI) (Catalog No. H1200, VECTASHIELD). The slides were subjected to fluorescence microscopy using a ZEISS microscope (Axio Imager Z2).

### Transfection

Cells were prepared in a 6-well plate and reached ~ 50% confluence before use. The siRNAs and reagents (Univ) were mixed in the buffer [Catalog No. 101000046, Univ (Shanghai, China)] for 10 min before transfection, with the final siRNA concentration being 50 nM/well. For assays where negative control (NC) was used, the control siRNA [Catalog No. AM4641, Invitrogen (California, America)] was transfected.

### Proliferation Assay

Cell viability was assessed by 3-(4,5-dimethylthiazol-2-yl)-2, 5-diphenyl-2H-tetrazol-3-ium bromide (MTT) assay (Catalog No. 298-93-1, Solarbio), following the manufacturer’s protocol. Cells in each well of a 96-well plate were supplemented with 10 *μ*l of MTT, 90 *μ*l of medium. After 4 h incubation at 37°C, the supernatant was discarded following the addition of 100 *μ*l of Dimethyl sulfoxide (DMSO) in each well for 10 min. Absorbance was detected using a microplate reader (Synergy H4). Student’s t-test was performed to evaluate the statistical significance with p < 0.05 being used as the cut-off.

### Co-Immunoprecipitation

Co-immunoprecipitation (Co-IP) was conducted following the manufacturer’s protocol (Catalog No. abs955, Absin).

Cells were incubated with a lysis buffer supplemented with protease and phosphatase inhibitors (Catalog No. P1010, Catalog No. CW2383, CWbio) for 10 min before they were washed by pre-cooled PBS. Cell lysates were placed on ice for 20 min, followed by centrifuge for 20 min. The supernatant was extracted and used for preparing the “Input” and immunoprecipitation (IP) samples.

The primary antibody was added to the cell supernatant for freeze rotation at 4°C overnight and added with agarose beads on the following day to prepare IP samples. Western blot was used to separate the samples.

## Results

### CAP Selectively Induces Autophagy in HCC Cells

**Figure 1:**
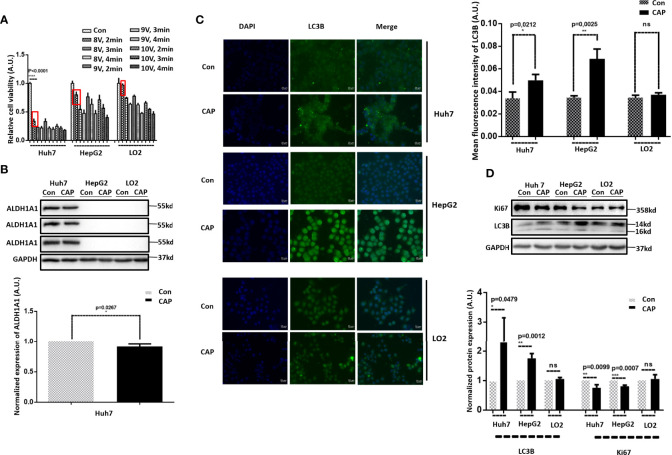
CAP selectively induces autophagy in HCC cells. **(A)** Relative cell viabilities of Huh7, HepG2, and LO2 cells under different CAP treatment doses. **(B)** Western blots and quantifications showing the expression of ALDH1A1 in Huh7, HepG2, and LO2 cells. **(C)** Western blots and quantifications showing the expression of LC3B in Huh7, HepG2, and LO2 cells with and without CAP treatment. **(D)** Immunofluorescence imaging and quantifications showing the level and location of LC3B in Huh7, HepG2, and LO2 cells with and without CAP treatment. 97L and 97H each represents MHCC97L and MHCC97H. Quantifications were made from triplicates. *, **, ***: statistical significance. ns: there is no statistical significance.

Before conducting in-depth phenotypic and mechanistic investigations, we first explored the parameter setting feasible for HCC treatment given the dose-dependent nature of CAP as an oncotherapeutic approach (24). By varying the CAP dosage, Huh7 showed higher sensitivity than HepG2 and LO2 cells to CAP treatment and exhibited little effect in LO2 cells under “8V supply voltage plus 2 min exposure duration,” which was selected as the parameter setting for CAP ejection in this study (**Figure 1A**). We next examined the cancer stemness level of the examined cell lines to help interpret experimental observations using ALDH1A1, a canonical marker of cancer stemness (25–27), as the molecular index. Among the three HCC lines, Huh7 had a high ALDH1A1 expression whereas the other two did not (p<0.001, **Figure 1B**), suggestive of a high cancer stemness of Huh7 cells. In addition, although slightly, CAP reduced the stemness of Hub7 cells with statistical significance (p=0.0267).

As autophagy has been lately shown to be capable of inhibiting cancer stemness in triple-negative breast cancers (28), and CAP was recently demonstrated with the attributes of triggering autophagy in melanoma cells (29); we examined whether and how CAP possibly could affect HCC cell autophagy.

IF and Western blot results showed that LC3B, a canonical marker of cell autophagy (30, 31), was substantially enhanced in Huh7 and HepG2 cells on CAP exposure, whereas that of LO2 cells was unaffected (**Figures 1C**, **D**), suggestive of a selective induction of autophagy in HCC cells as compared with normal liver cells. We also noted that CAP slightly halted the proliferation of Huh7 and HepG2 cells with statistical significance without altering that of LO2 (KI67, **Figure 1D**), suggesting the selective cytotoxicity of CAP against malignant liver cells.

### CAP Selectively Affects Acetylation in HCC Cells

**Figure 2:**
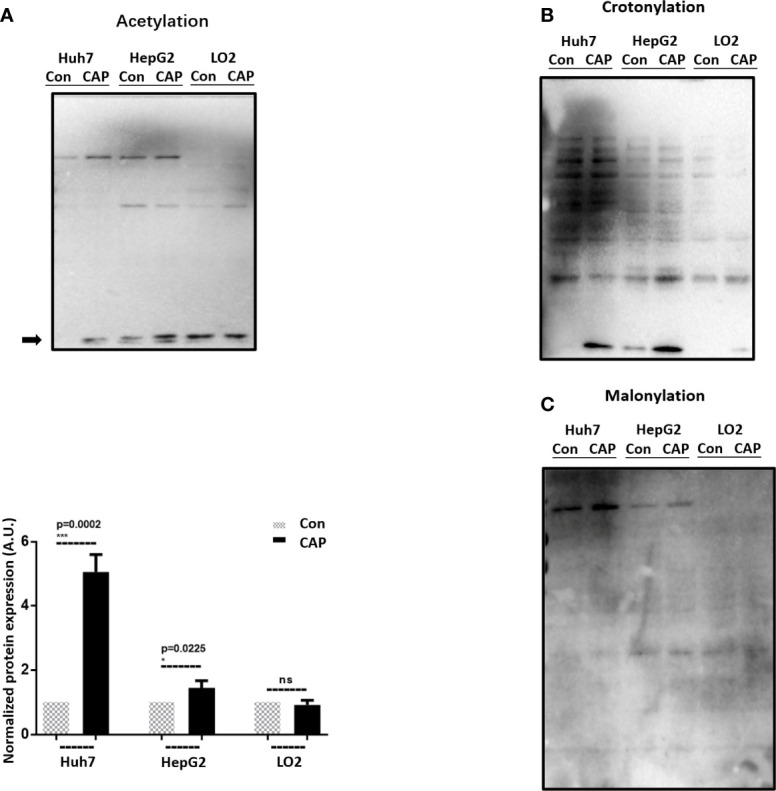
CAP selectively affects acetylation in HCC cells. Western blots and quantifications showing the expression profiles of **(A)** acetylation, **(B)** crotonylation, and **(C)** myristoylation in Huh7, HepG2, and LO2 cells with and without CAP treatment. Quantifications were made from triplicates for panel **(A)**. *, ***: statistical significance. ns: there is no statistical significance.

Post-translational modifications (PTMs) including oxidation have been proposed with prominent roles in autophagy regulation (32, 33). With rapid technology advancement, novel PTM programs keep being discovered, among which acetylation, crotonylation, and myristoylation were examined here before and after CAP treatment. Following the order of cell malignancy from high to low (Huh7, HepG2, LO2), we found one lane (indicated by an arrow) in the acetylation profile that was elevated after CAP treatment in cancer cells (Huh7, HepG2) and the basal level (control) of which decreased with cell malignancy (**Figure 2A**), implicative of its tumor-suppressive role and the efficacy of CAP in restoring it back to the normal level. Though CAP enhanced the level of one lane in the crotonylation profiles of Huh7 and HepG2 cells, its basal expression was similar between Huh7 and LO2 cells, which was exempted from the analysis here (**Figure 2B**). Although one lane in the myristoylation profiles was enhanced in Huh7 and HepG2 cells but not in LO2 cells, its level was positively associated with cell cancer stemness, yet CAP showed an inductive role, removing it from our focus here (**Figure 2C**). Thus, we considered acetylation as the most relevant PTM in response to CAP treatment, among the examined PTM events, and selected it for the following investigations. In particular, we used Huh7 cells in the following experimental assays given its highest CAP sensitivity among all tested cell lines.

### CAP Triggers Autophagy in HCC Cells *via* Suppressing EGFR Acetylation and EGFR(Tyr1068) Phosphorylation

Inspired by the critical roles of EGFR reported in reactive oxygen species (ROS)-triggered autophagy in non-small cell lung cancer cells (34), we focused on the possible involvement of EGFR in CAP-induced autophagy and acetylation alteration.

**Figure 3:**
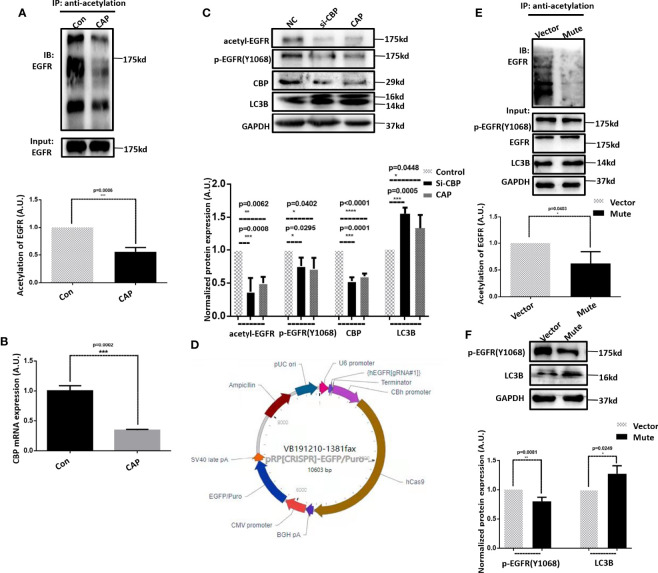
CAP triggers autophagy in HCC cells *via* suppressing EGFR acetylation and EGFR(Tyr1068) phosphorylation. **(A)** Immunoprecipitation and its quantification showing EGFR acetylation with and without CAP treatment. **(B)** Q-PCR results showing the knockdown efficiency of CBP. **(C)** Western blots and quantifications showing the level of EGFR acetylation, EGFR(Tyr1068) phosphorylation, and LC3B. **(D)** Plasmid structure generating EGFR(Tyr1068) mutation. **(E)** Immunoprecipitation and its quantification showing EGFR acetylation with and without EGFR(Tyr1068) mutation. **(F)** Western blots and quantifications showing the level of EGFR(Tyr1068) and LC3B. Quantifications were made from triplicates. *, **, ***, ****: statistical significance. ns: there is no statistical significance.

Indeed, CAP substantially reduced EGFR acetylation (**Figure 3A**). Silencing *CBP*, an EGFR acetyltransferase (35), using sequences obtained from (36) (p=2E-4, **Figure 3B**) or treating cells with CAP both reduced EGFR acetylation and EGFR(Tyr1068) phosphorylation levels and enhanced LC3B expression (**Figure 3C**), suggesting the role of CAP in suppressing EGFR acetylation and EGFR(Tyr1068) phosphorylation as well as the involvement of both PTMs in CAP-triggered autophagy.

Using the CRISPER/Cas9 technology, we constructed the EGFR(Tyr1068) mutant by mutating the tyrosine 1068 site to phenylalanine that blocks EGFR phosphorylation at the 1068 site (**Figure 3D**). The acetylation of the EGFR(Tyr1068) mutant was remarkably reduced (**Figure 3E**), suggestive of a positive association between EGFR(Tyr1068) phosphorylation and acetylation as well as their interactions. The LC3B level was elevated in the EGFR(Tyr1068) mutant (**Figure 3F**), further supporting the suppressive role of EGFR(Tyr1068) phosphorylation in HCC autophagy.

### EGFR(Tyr1068) Phosphorylation Affects LC3B Ubiquitination

**Figure 4:**
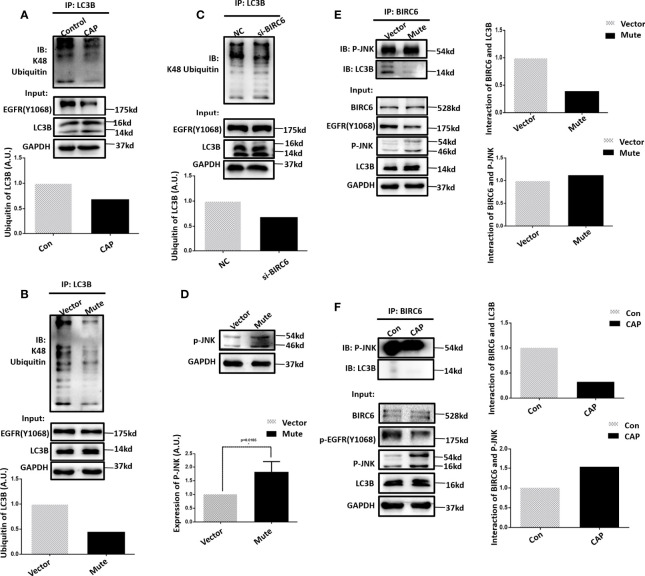
EGFR (Tyr1068) phosphorylation affects LC3B ubiquitination. **(A)** Immunoprecipitation and its quantification showing LC3B K48 ubiquitination with and without CAP treatment. **(B)** Immunoprecipitation and its quantification showing LC3B K48 ubiquitination with and without EGFR(Tyr1068) mutation. **(C)** Immunoprecipitation and its quantification showing LC3B K48 ubiquitination with and without silencing BIRC6. ‘NC’ refers to cells transfected with control siRNA. **(D)** Western blot and quantification showing the level of phosphorylated JNK (p-JNK). **(E)** Immunoprecipitation and its quantification showing interactions between p-JNK and BIRC6, LC3B, and BIRC6 with and without EGFR(Tyr1068) mutation. Quantifications were made from triplicates. (**F**) Immunoprecipitation and its quantification showing interactions between p-JNK and BIRC6, LC3B, and BIRC6, receiving or not receiving CAP treatment. *: statistical significance.

**Figure 5:**
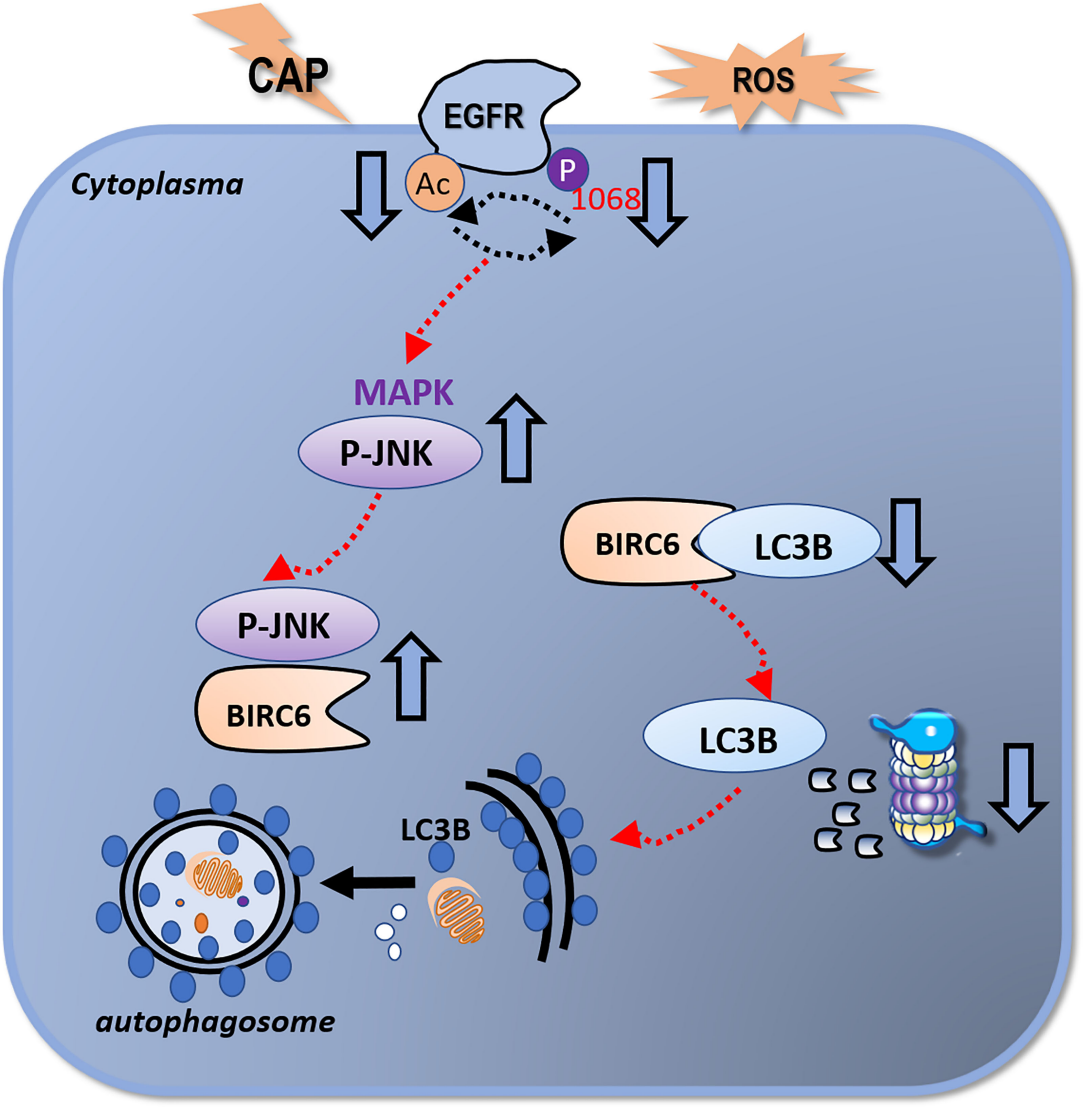
Illustrative diagram on the mechanism of CAP in triggering autophagy in HCC cells. CAP suppresses EGFR acetylation and EGFR(Tyr1068) phosphorylation that interfere with each other and collectively lead to an elevated level of p-JNK as well as its enhanced interactions with BIRC6. This reduces the availability of BIRC6 in interacting with LC3B, which results in reduced LC3B ubiquitination and enhanced autophagy.

We next explored the potential molecular mechanism that drives the mediating role of EGFR(Tyr1068) on cell autophagy in response to CAP treatment. It was demonstrated that the observed elevated level of LC3B (**Figure 3C**) after CAP treatment was a result of reduced LC3B ubiquitination (**Figure 4A**), and blocking EGFR(Tyr1068) phosphorylation suppressed LC3B K48 ubiquitination (**Figure 4B**). In addition, silencing *BIRC6*, an E3 ubiquitin ligase of LC3B (37), slightly decreased LC3B K48 ubiquitination (**Figure 4C**), and mutating EGFR(Tyr1068) enhanced phosphorylated JNK (p-JNK) (**Figure 4D**) that was known to interact with BIRC6 (38), elevated interactions between BIRC6 and p-JNK, and decreased interactions between BIRC6 and LC3B (**Figure 4E**). CAP treatment showed a similar effect with EGFR(Tyr1068) mutant in enhancing the interaction between BIRC6 and p-JNK and decreasing that between BIRC6 and LC3B (**Figure 4F**). These collectively suggested a competition between p-JNK and LC3B in binding with BIRC6. That is, CAP reduced EGFR acetylation and EGFR(Tyr1068) phosphorylation, which led to elevated p-JNK as well as its interactions with BIRC6; this resulted in decreased availability of BIRC6 in interacting with LC3B, reduced LC3B ubiquitination, and consequently, an enhanced level of autophagy (**Figure 5**).

## Discussion

We reported in this study the selectivity of CAP against HCC cells by triggering autophagy and identified the EGFR/p-JNK/BIRC6/LC3B axis in driving this process. Importantly, we unveiled the critical and orchestrated roles of multiple PTM events in this molecular axis that involves EGFR acetylation, EGFR(Tyr1068) phosphorylation, and LC3B ubiquitination.

Autophagy is a process that degrades autologous protein and damaged organelles (39) that may prevent cancer cells from spontaneous hyper-proliferation *via* arresting them at the G_0_ phase or even at the death state if excessive, providing the reasoning behind the selectivity of CAP against the HCC cells identified in this study.

LC3B, a protein involved in the formation of autophagosomes, has been widely used as a marker of autophagy (31, 40). We found from our assays that LC3B sometimes showed two stripes and sometimes one stripe. The antibody we used (Catalog No. 83506S, Cell Signaling Technology) was capable of identifying both LC3B-II and LC3B-I. During autophagy, LC3B-I is gradually transformed into lipid LC3B-II, and LC3B-I is less stable and easily degraded during repeated freezing and thawing (40). Thus, the inconsistency regarding the number and intensities of the stripes of LC3B observed was mainly caused by the differential autophagy stages measured in each assay, as well as the differential sample storage duration and condition. Crosstalk among the different types of PTMs during disease initiation and development has been frequently reported and gaining increasing attention (41–47). Here, we reported the collective roles of EGFR acetylation and phosphorylation in determining LC3C ubiquitination. We found a reciprocal relationship between EGFR acetylation and phosphorylation but did not explore their causal relationship. That is, whether CAP triggered EGFR acetylation first that led to EGFR(Tyr1068) phosphorylation, or the other way around, or CAP induced EGFR acetylation and EGFR(Tyr1068) phosphorylation simultaneously was unknown and left for further investigations. In addition, we did not explore the activity and possible roles of other EGFR phosphorylation sites such as Tyr992, Tyr1086, Tyr1148, and Tyr1173 in CAP-triggered HCC autophagy, which warrant additional studies. Autophagy can help halt cancer cell growth; it may also protect cells from oxidative damage if occurring under the physiological condition. In other words, autophagy may confer a favorable value to normal cells under the physiological condition (48–50). We found from our studies that given a higher dose, CAP could also trigger autophagy in normal liver cells (**Figure 1B**), implicative of its beneficial roles to human health. Liver is the main organ that functions in detoxification. Triggering its self-renewal *via* autophagy is compatible with the eternal pursuit of human beings for longevity and rejuvenation and is relevant to protecting liver against alcohol hangover. These make the clinical translation of CAP more interesting and promising that, once holds true, renders CAP not only a potential novel onco-therapeutic strategy but also a health maintenance tool.

Lastly, it is worthy to mention that the nuclei of HepG2 cells seemed to be larger after CAP treatment (**Figure 1C**), implicative of the possible existence of other CAP-triggered cellular outcomes that are beyond the scope of this study and deserve additional investigations.

## 5 Conclusions

We reported the efficacy of CAP in selectively arresting the growth of HCC cells *via* triggering autophagy and proposed the EGFR/p-JNK/BIRC6/LC3B molecule axis that drove this process. We emphasized the importance of multiple PTM orchestration in this molecular axis that involved EGFR acetylation, EGFR(Tyr1068) phosphorylation, and LC3B ubiquitination. Our study unveiled the selectivity of CAP against HCC cells and also forecasted its utilities in a broader scope including, for example, longevity and anti-aging, as a promising tool for human health maintenance.

## Data Availability Statement

The original contributions presented in the study are included in the article, further inquiries can be directed to the corresponding author.

## Author Contributions

XD conceptualized the idea, supervised this study, and drafted the manuscript. DW and LC conducted the experiments and prepared the figures and tables. JZ provided a partial financial support and co-supervised this study. All authors have read and approved the final draft.

## Funding

This study was funded by the National Natural Science Foundation of China (Grant No. 81972789), Fundamental Research Funds for the Central Universities (Grant No. JUSRP22011), and Technology Development Funding of Wuxi (Grant No. WX18IVJN017). These funding sources have no role in the study design, data collection and analysis, decision to publish, or preparation of the manuscript.

## Conflict of Interest

Author XD was employed by CAPsoul Medical Biotechnology Company, Ltd.

The remaining authors declare that the research was conducted in the absence of any commercial or financial relationships that could be construed as a potential conflict of interest.

## Publisher’s Note

All claims expressed in this article are solely those of the authors and do not necessarily represent those of their affiliated organizations, or those of the publisher, the editors and the reviewers. Any product that may be evaluated in this article, or claim that may be made by its manufacturer, is not guaranteed or endorsed by the publisher.

## Supplementary Material

Supplementary Table 1 Information on the siRNAs used in this study.

Supplementary Table 2 Information on primers used in this study.
